# Modulation of Ras/ERK and Phosphoinositide Signaling by Long-Chain n-3 PUFA in Breast Cancer and Their Potential Complementary Role in Combination with Targeted Drugs

**DOI:** 10.3390/nu9030185

**Published:** 2017-02-23

**Authors:** Simona Serini, Gabriella Calviello

**Affiliations:** Institute of General Pathology, Università Cattolica del S. Cuore, Largo F. Vito 1, 00168 Rome, Italy; simona.serini@unicatt.it

**Keywords:** breast cancer, cytotoxic effect, docosahexaenoic acid, eicosapentaenoic acid, omega-3 PUFA, phosphoinositide pathway, ERK pathway, in vitro studies, animal studies, human studies

## Abstract

A potential complementary role of the dietary long-chain n-3 polyunsaturated fatty acids (LCn-3 PUFA) in combination with innovative mono-targeted therapies has recently been proposed. These compounds are thought to act pleiotropically to prevent the development and progression of a variety of cancers, including breast cancer. We hereinafter critically analyze the reports investigating the ability of LCn-3 PUFA to modulate the Ras/ERK and the phosphoinositide survival signaling pathways often aberrantly activated in breast cancer and representing the main targets of innovative therapies. The in vitro or in vivo animal and human interventional studies published up to January 2017 investigating the effects of LCn-3 PUFA on these pathways in normal and cancerous breast cells or tissues were identified through a systematic search of literature in the PubMed database. We found that, in most cases, both the in vitro and in vivo studies demonstrated the ability of LCn-3 PUFA to inhibit the activation of these pro-survival pathways. Altogether, the analyzed results strongly suggest a potential role of LCn-3 PUFA as complementary agents in combination with mono-targeted therapies. Moreover, the results indicate the need for further in vitro and human interventional studies designed to unequivocally prove the potential adjuvant role of these fatty acids.

## 1. Introduction

Several in vitro and in vivo experimental studies have supported the antineoplastic role of the long-chain n-3 polyunsaturated fatty acids (LCn-3 PUFA) eicosapentaenoic acid (EPA) and docosahexaenoic acid (DHA), present at high levels in fatty fish. One remarkable feature of these dietary compounds and their metabolic derivatives is the ability to act pleiotropically to prevent the development and progression of cancer [1,2]. Indeed, they have been shown to reduce inflammation and induce its resolution [3], as well as to foster apoptosis [4] and differentiation [5] in cancer cells. Moreover, they have been reported to inhibit cancer cell proliferation, migration, invasion, neo-angiogenesis, and metastasis [6]. Multiple components of molecular pathways involved in these processes are known to be targets of LCn-3 PUFA action that can significantly affect their expression and/or activity [7]. Among these targets are membrane receptors, protein kinases and phosphatases [8,9], transcription factors, cyclins, and components or modulators of the apoptotic and autophagic pathways [10]. In the era of innovative targeted anti-cancer therapy, agents such as LCn-3 PUFA, which lack specificity and are able to simultaneously affect a variety of targets, should be regarded with caution. However, although multiple and often unrelated, their effects at molecular levels are indisputably powerful. According to an accepted hypothesis, all these effects may be correlated to the ability of fatty acids to powerfully modify the properties of the cell membrane environment. This hypothesis suggests that the enrichment of the membrane with these fatty acids may deeply change the cellular fatty acid profile, particularly at the expense of n-6 PUFA, thus leading to a reduced production of pro-inflammatory and pro-carcinogenic omega-6 derivatives [7]. Moreover, it was suggested that the increased incorporation of LCn-3 PUFA has the potential to deeply alter the physicochemical properties of membranes, in particular the lipid membrane microenvironments (rafts) where signaling molecules (carrier, enzymes, receptors) are assembled and facilitated in their correlations [11]. As a matter of fact, it has been observed that the incorporation of LCn-3 PUFA in membranes alters the expressions of several signaling molecules residing there, and, in turn, modifies the activities of factors/pathways placed downwards [12,13]. Indeed, it has been suggested [14,15] that, thanks to their pleiotropic anticancer properties, LCn-3 PUFA could represent optimal candidates for adjuvant cancer therapy, in combination with conventional drugs as well as targeted therapies. As a matter of fact, some of the innovative anti-cancer therapies have failed due to their frequent and dangerous side effects, as well as to the multilevel cross stimulations occurring among the targets of these new biological agents. This means that the specific suppression of one pathway often induces alternative pathways that may function as savage or escape mechanisms, and induce resistance in cancer cells [16]. The possibility to combine more than one targeted agent has recently been suggested in order to suppress multiple signaling pathways and overcome resistance. The simultaneous treatment of mono-targeted drugs with multi-targeted dietary agents, such as LCn-3 PUFA, could also represent an interesting alternative option [14,17,18]. In particular, for their properties, these fatty acids do not induce any dangerous side effect at the doses generally used in patients, in addition to further improving the activities of the mono-targeted therapies.

**Figure 1:**
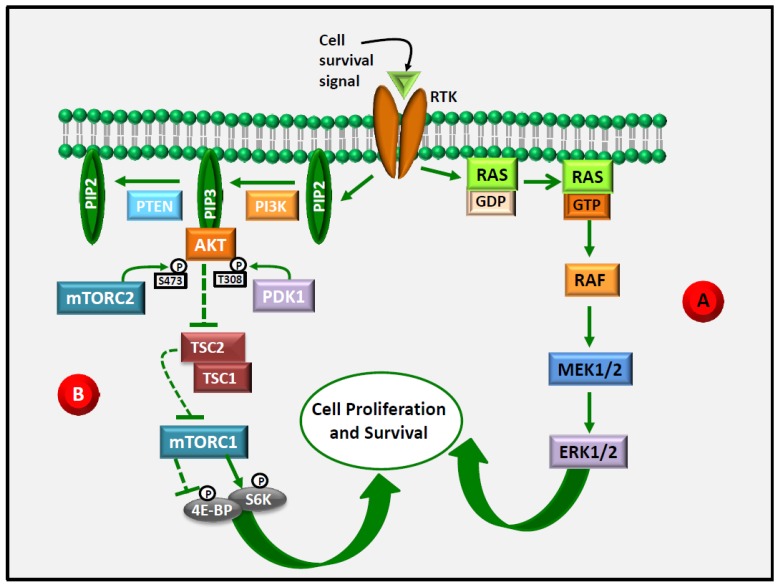
Activation of the RAS/ERK and phosphoinositide cell signaling pathways following the binding of cell survival signals to receptor tyrosine kinases (RTK). The two pathways are triggered by the activation of RTK in the cell membrane. After the binding of RTK by cell survival signals, the RTK intracellular domain is phosphorylated and (**A**) through several steps it allows the binding and activation of RAS. Consequently, RAF, the first MAPK in this pathway, is recruited and activated and, in turn, it phosphorylates and activates MEK1/2, which finally activates ERK1/2 by the dual phosphorylation on tyrosine and threonine. In turn, ERK activates a variety of substrates placed downwards that ultimately induce cell proliferation and survival; (**B**) PI3K is associated with the intracellular domain of RTK and activated to catalyze the PIP2 phosphorylation into PIP3 that recruits AKT. In this form, AKT can be activated by the dual phosphorylation carried out respectively by PDK1 and mTORC2. Activated AKT regulates the activities of downstream targets that ultimately lead to the induction of cell proliferation and survival. PTEN may regulate this pathway by dephosphorylating PIP3. See the text for more details regarding the components and reactions of the two signaling pathways.

This review will examine the ability of LCn-3 PUFA to modulate the activation of two signaling pathways: the Ras/ERK pathway (also named mitogen-activated protein kinase (MAPK)/ERK or the Ras/Raf/MEK/ERK pathway) and the phosphoinositide pathway (also named the phosphatidylinositol 3-kinase (PI3K)/Akt/mTOR pathway) (Figure 1). In recent years, the components of these pathways have been specifically targeted by drugs designed to bind/inhibit them with highly variable success rates. The two pathways share essential roles in the regulation of cell cycle progression and cell survival, and their aberrant activation has been involved in the development and progression of a variety of malignancies.

## 2. Methods

A systematic review of the literature in the PubMed database was performed to identify peer-reviewed original research articles published (both online or in print) in the English language during the period January 2005–January 2017, and regarding in vitro and in vivo animal studies, as well as human interventional studies investigating the modulatory effects of the two LCn-3 PUFAs EPA and DHA on the ERK and phosphoinositide survival pathways in normal and cancerous breast cells or tissues.

**Figure 2:**
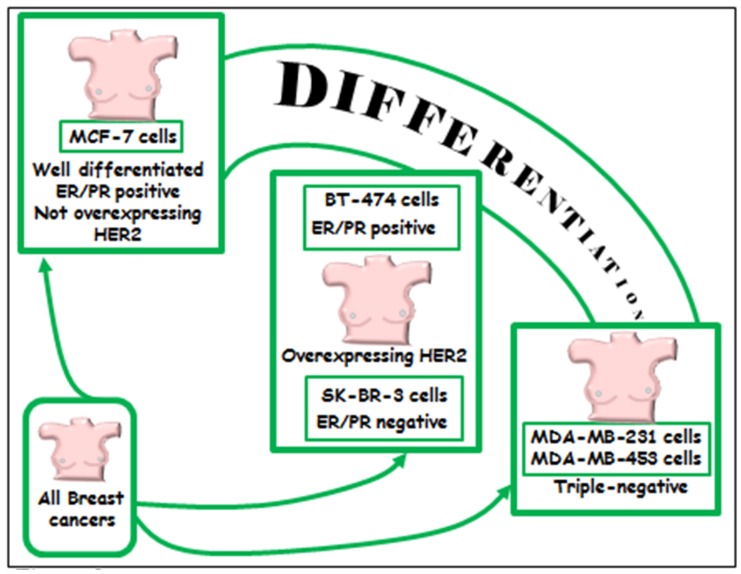
Outline of the breast cancer cell lines used in most of the in vitro studies analyzed, and their main features. MCF7 cells are the most differentiated and represent the most used in vitro model for well-differentiated breast cancer, since they express Estrogen Receptors (ER), and/or Progesteron Receptors (PR). Both ER/PR positive BT-474 and ER/PR negative SK-BR-3 cells are scarcely differentiated cells and share the overexpression of the receptor tyrosine kinase HER2 codified by the oncogene ErbB2. They represent in vitro models for HER2 positive breast cancer. MDA-MB-231 and MDA-MB-453 cells are the least differentiated, and represent in vitro models for triple negative breast cancers, i.e., they do not express ER/PR receptors or overexpress HER.

**Figure 3:**
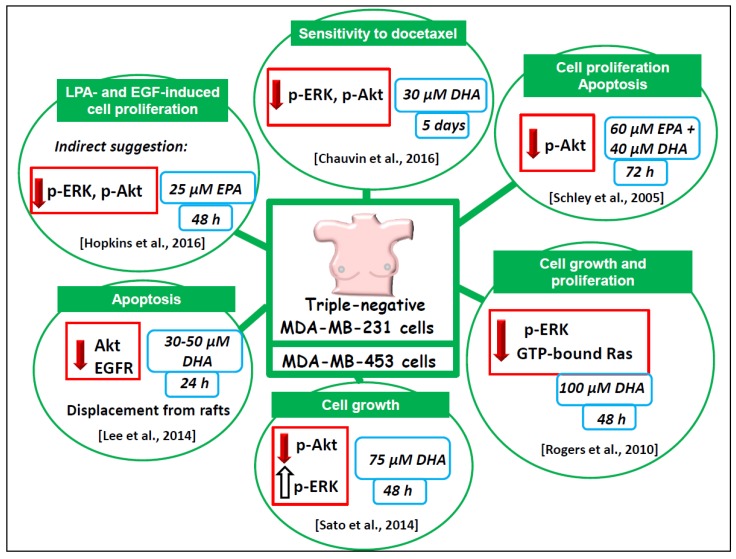
Outline of the main results of the in vitro reports investigating LCn-3 PUFA regulation of ERK, AKT and their upstream components in the ERK and phosphoinositide survival pathways in triple negative breast cancer cells. The green labels show the main features, functions, or cellular processes that are related to cell growth and regulated in an antineoplastic manner by LCn-3 PUFA in MDA-MB-231 and MDA-MB-453 breast cancer cells. The red arrows indicate a negative regulation by LCn-3 PUFA, and the empty arrow indicates activation. The blue boxes illustrate the LCn-3 PUFA concentrations used and the duration of incubation with fatty acids.

**Figure 4:**
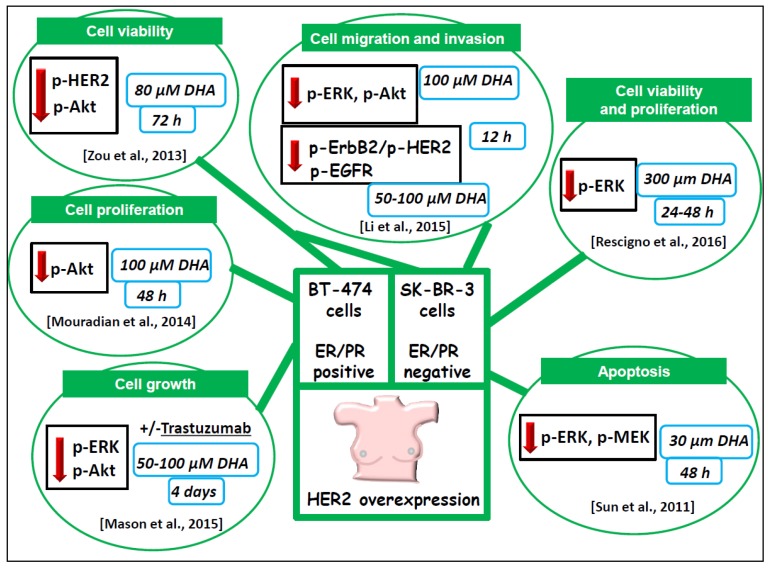
Outline of the main results of the in vitro reports investigating LCn-3 PUFA regulation of ERK and AKT and their upstream components in the ERK and phosphoinositide survival pathways in BT-474 and SK-BR-3 breast cancer cells. The green labels show the main features, functions, or cellular processes that are related to cell growth and regulated in an antineoplastic sense by LCn-3 PUFA in these breast cancer cells. The red arrows indicate negative regulation by LCn-3 PUFA. The light blue boxes illustrate the LCn-3 PUFA concentrations used and the length of the incubations with fatty acids.

**Figure 5:**
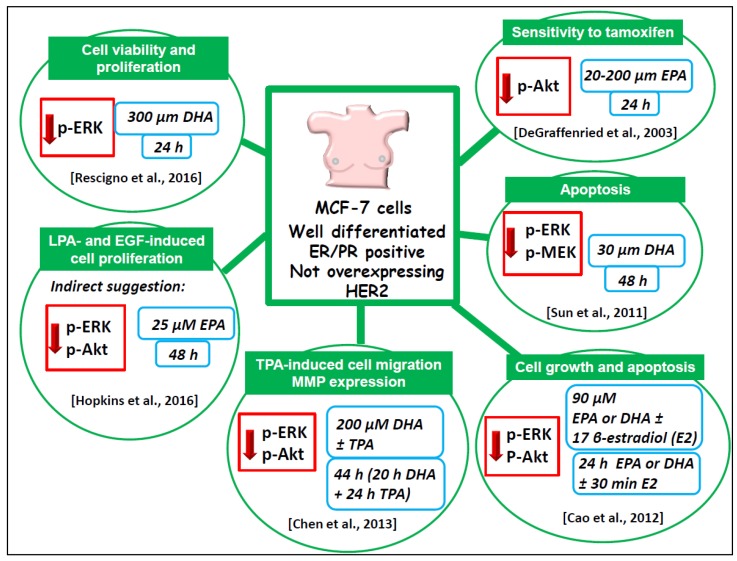
Outline of the main results of the in vitro reports investigating LCn-3 PUFA regulation of ERK and AKT and their upstream components in the ERK and phosphoinositide survival pathways in MCF7 breast cancer cells. The green labels show the main features, functions, or cellular processes that are related to cell growth and regulated in an antineoplastic sense by LCn-3 PUFA in these breast cancer cells. The red arrows indicate negative regulation by LCn-3 PUFA. The light blue boxes illustrate the LCn-3 PUFA concentrations used and the length of incubations with fatty acids.

**Figure 6:**
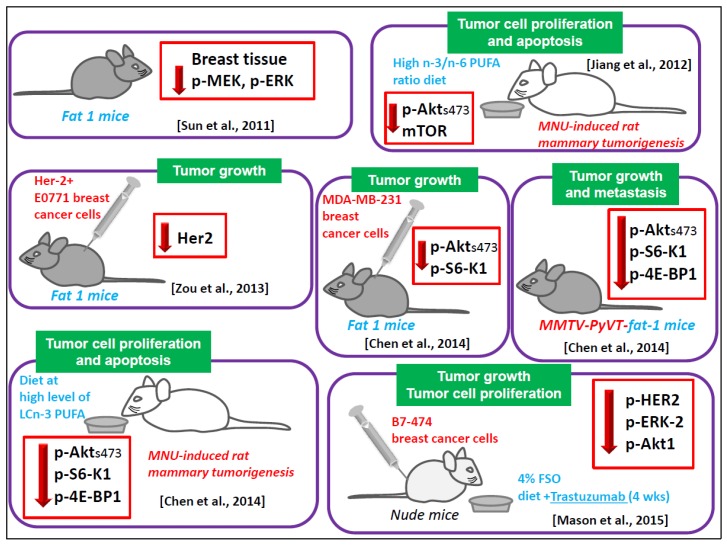
Outline of the main results of the reports investigating LCn-3 PUFA regulation of ERK and AKT and their upstream or downstream components in the ERK and phosphoinositide survival pathways in in vivo animal models. The green labels show the main features, functions, or processes of the tumors/breast cancer cells growing in vivo and regulated in an antineoplastic sense by LCn-3 PUFA. The red arrows indicate a negative regulation by LCn-3 PUFA. The red captions explain the treatment performed or the animal models used to obtain the development of breast tumors in the animals. The light blue captions describe the treatment or animal models used to obtain animals with LCn-3 PUFA enriched tissues and cells. *MMTV-PyVT-fat-1 mice:* transgenic mice obtained by crossing Fat-1 mice with polyoma virus middle T oncogene mammary tumorigenesis model (MMTV-PyVT) mice.

The followings terms in the title or abstract were used in the research: omega-3/n-3 PUFA/DHA/EPA and MAPK/ERK/RAS/RAF/PI3K/AKT/mTOR and cancer/breast cancer and in vitro studies/in vivo studies/animal studies and human/clinical trials. The literature analysis was carried out independently by both the authors, and a worksheet was created including the outcomes (both the main and additional ones) of all the retrieved studies. We widely discussed the existing bias of some of the analyzed studies throughout Section 6. The human breast cancer cell lines used in most of the in vitro studies analyzed and their main features are described in Figure 2. The results of our study analysis performed in human breast cancer cells in vitro are summarized in Figure 3, Figure 4 and Figure 5. In particular, Figure 3 reports the results related to the studies carried out on the triple-negative MDA-MB-231 and MDA-MB-453 cells, Figure 4 illustrates those concerning the studies performed in the HER2 over-expressing ER/PR positive BT-474 and ER/PR negative SK-BR-3 cells. Figure 5 reports the results related to the studies performed on the well-differentiated ER/PR positive MCF-7 cells not expressing HER2. The results regarding the studies performed using breast cancer animal models are summarized in Figure 6.

## 3. ERK1/2 as an Emerging Target in Anti-Cancer Therapy

The mitogen-activated protein kinase (MAPK) cascade, is the best characterized of the four conventional subfamilies of MAPK cascades transducing a large number of signals, and that lead, as the last step, to the activation of four different MAPK or groups of MAPK: ERK1/2; JNK 1,2 and 3; p38 α, β, γ and δ; and the recently discovered ERK5 [19]. As soon as they are activated, in turn, they phosphorylate a large variety of substrates involved in well-established cell responses, including cell proliferation, differentiation, apoptosis, and migration [19]. The RAS/ERK signaling pathway is generally activated following the binding of ligands to a wide range of membrane receptors whose activation, through several steps, will lead to the binding and activation of RAS to GTP. This will result in the recruitment and activation of the first MAPK in the pathway (named for its upward role MPKKK), i.e., in this case B-RAF and C-RAF, followed by the phosphorylation of the MEKK MEK1/2, which lastly activates the MAPK ERK1/2 by the dual phosphorylation on tyrosine and threonine [20]. Complex controls and feedback mechanisms regulate the correct functioning of the RAS/RAF/MEK/ERK1/2 cascade, given the essential role played by this signaling pathway in the modulation of cell growth and survival. On the other hand, its abnormal activation has been involved in the development and progression of almost one-third of all human cancers [21]. In particular, in most cases mutations in BRAF or RAS (KRAS, HRAS, or NRAS) have been detected. Interestingly, it is worth noticing that these two classes of mutations are mutually exclusive in malignancies. While, on one hand, this observation indicates that just one of them can be enough to dysregulate the entire Ras/Raf/MEK/ERK1/2 cascade, on the other hand it further supports the critical role played by the abnormal activation of this cascade in carcinogenesis. As analyzed in detail in a recent review by Wu and Park [22], RAS mutations are detected in more than half of the pancreas cancers, and in about a third of colon, biliary tract, and skin cancers, as well as in around a fifth of small intestine or lung cancers. A series of other cancers (ovary, salivary glands, urinary tract, cervix, endometrium, upper aero-digestive tract, prostate, thyroid, and hemopoietic/lymphoid cells) [22] are characterized by RAS mutations, although with slightly lower proportions (ranging from 14% to 18%). Also, mutations in BRAF, particularly those involving the Valine600 codon, are found at very high percentages in several cancers. Whereas, for instance, 100% of hairy cell leukemia is characterized by this mutation, it has been detected in 50%–70% of melanomas, 57% of Langerhans cell histiocytosis, 40% of papillary thyroid cancers, and more than 30% of low-grade ovarian carcinomas [22]. Given the wide diffusion and the role played by these mutations in a large number of malignancies, great efforts are being made in the development of drugs specifically targeting members of the RAS/RAF/MEK/ERK1/2 cascade. Whereas direct RAS targeting has not allowed us to obtain a drug suitable for human use [23], in recent years a series of RAF and MEK specific inhibitors has been developed against several kinds of Ras/Raf/MEK/ERK-driven cancers [23]. They have shown considerable clinical efficacy, although patients generally experience acquired resistance after some months of therapy. Interestingly, in many cases the resistance was acknowledged as an ERK-dependent resistance. This implies that, despite the innovative targeted therapy with BRAF and MEK inhibitors, ERK1/2 may represent the weak link in this system, since, unless controlled by a therapy directly targeting it, ERK1/2 can still activate the substrate placed downwards. This has been ascribed either to a mutation of key regulators (e.g., BRAF, MEK1, MEK2), able in the mutant form to escape the targeted therapy, or to the lack of their feedback negative regulation by ERK1/2. On this basis, it has been suggested that the direct use of ERK inhibitors could overcome acquired resistance to BRAF and MEK inhibitors. Their use appears to be particularly advantageous, since mutations of ERK1/2 able to induce acquired resistance have not been described [24].

Significant progress has been made in order to understand the mechanisms of resistance that often arise during the administration of therapy with inhibitors of the Ras/Raf/MEK/ERK pathway and other signaling pathways abnormally activated in cancer. Moreover, innovative strategies are being proposed to overcome the resistance and improve the efficacy of the current targeted treatments for a variety of cancers. The research in this area is continuously leading to the discovery of new inhibitors for this and other signaling pathways, or the discovery of novel therapeutic targets among the regulators/components of the Ras/Raf/MEK/ERK and other signaling pathways. Moreover, new therapies are also being experimented by combining different targeted inhibitors or combining targeted inhibitors with other existing non-targeted anticancer agents. In particular, recent studies have addressed the possibility of combining anticancer targeted therapies with natural agents, known for their broad antineoplastic activities, and relatively low toxicity, including LCn-3 PUFA fatty acids [25,26].

## 4. Akt as an Emerging Target in Anti-Cancer Therapy

Plenty of evidence has demonstrated that LCn-3 PUFA fatty acids may induce antineoplastic effects also by inhibiting the phosphoinositide signaling in cancer cells [9,27,28]. Interestingly, particular attention has been given to the combination between the therapies simultaneously targeting the RAS/ERK and the phosphoinositide pathways in the search of novel combination strategies to overcome resistance and extend the time-limited responses to therapy against members of the Ras/ERK pathways. The latter signaling system is a further well-known driver of oncogenic activity in human malignancies, as frequent mutations affect its components, thus leading to its aberrant activation. This, in addition to contributing to the development and progression of several forms of cancers, has often been associated with resistance to chemotherapy and targeted therapies, including those directed against the Ras/ERK1/2 pathway [29,30,31,32].

The phosphoinositide pathway (Figure 1) is often referred to as a “survival” pathway, as, among other functions, in normal cells it plays an essential role in the induction of cell cycle progression and the inhibition of apoptosis. PI3K is a group of lipid kinases which phosphorylate phosphatidylinositol 4,5-disphosphate (PIP2) to phosphatidylinositol 3,4,5-trisphosphate (PIP3), an important intracellular signaling molecule involved in the regulation of cell growth, proliferation and survival, and whose aberrant activation often represents a crucial step in carcinogenesis. There are four classes of PI3K, differently expressed in different kinds of cancers. Class I PI3K (further subdivided in class Ia and Ib) is the most implicated in the pathway activated in response to the growth-factor (GF) binding to receptor protein tyrosine kinases (RTK), and in the development of cancer. The binding of several RTK by specific cell survival signals activates the phosphoinositide pathway, triggering the association of PI3K with the intracellular domain of RTK. As PIP3 is generated, it interacts with specific domains of a number of proteins, including Akt. Three different isoforms of Akt (Akt1, Akt2, and Akt3) are encoded by three different genes (for a review, see [33]). As Akt is transiently recruited by PIP3 to the plasma membrane, it modifies its conformation, thus allowing phosphoinositide-dependent kinase 1 (PDK 1) to phosphorylate threonine 308 (T308) in its catalytic domain. In order to be fully activated, Akt requires an additional phosphorylation on the serine 473 (S473) present in its carboxy-terminal domain, which is carried out by the activated form of the mammalian target of rapamycin (mTOR) complex2 (mTORC2). This enzymatic complex is composed of the serine/threonine kinase mTOR which binds to the following proteins: rapamycin-insensitive companion of mTOR (Rictor); mammalian stress-activated protein kinase interacting protein (mSIN1); protein observed with Rictor-1 (Protor-1); mammalian lethal with Sec 13 protein 8 (mLST8, also known as GβL); and DEP-domain-containing mTOR-interacting protein (Deptor) [34,35]. In turn, fully activated Akt phosphorylates and inactivates tuberous sclerosis complex2 (TSC2), thus promoting the proteasomal degradation of the TSC1/TSC2 complex, which acts as a negative regulator of the mTORC1 complex, thus leading to mTORC1 activation. Similarly to mTORC2, mTORC1 is a multiprotein complex including the proline-rich AKT substrate 40 kDa (PRAS40), a component that is not found in mTORC2, as well as other components also shared by mTORC2 (mTOR, Raptor, mLST8, Deptor) [34,35]. Various signals, growth factors, energy statuses, and amino acids are able to induce the activity of mTORC1 [35]. Activated mTORC1 subsequently phosphorylates eIF4E binding proteins (4E-BP) and ribosomal S6 kinases (S6K), thus inducing mRNA translation and protein synthesis. In normal conditions, Akt activity is under the control of protein phosphatases (PPA2A and PHLPP2) that directly dephosphorylate it, as well as of the tumor suppressor phosphatase and tensin homolog (PTEN), which dephosphorylates PIP3, thus indirectly inhibiting Akt activation.

Melanoma and cancers arising in the lungs, breast, endometrium, cervix, ovaries, thyroid, gastrointestinal tract, and pancreas are among the tumors that have been reported to show Akt overexpression or activation. The overexpression has very often been related to activating point mutations and to the amplification of PIK3, mutation of PTEN leading to its loss of function, PTEN epigenetic silencing [36,37], or to the amplification of Akt gene, and post-translational modification of Akt protein. On the other hand, very rarely Akt mutations, affecting one of the three AKT isoforms, are found in tumors [33,38]. The use of drugs targeting growth factor receptors (EGFR, HER2) placed upstream of the phosphoinositide signaling pathway has often resulted in resistance due to the phosphorylation/activation of Akt through alternative compensatory pathways [33,39,40]. Moreover, targeting mTORC1, placed downstream of Akt, was reported to be unsuccessful since, as a result of the disappearance of the feedback mTORC1-induced Akt inhibition, Akt activity may even result to be increased [33,38]. Furthermore, the specific inhibition of PI3K has not yielded satisfactory responses but rather dangerous side effects [41]. For these reasons, the possibility of directly inhibiting Akt itself has recently gained increasing interest [33]. Different promising drugs targeting Akt have been developed and extensively investigated in the preclinical setting, and several clinical trials are now underway to assess the efficacy and safety of Akt inhibitors in different kinds of cancer [42,43].

## 5. ERK1/2 and Akt Are Central Targets in LCn-3 PUFA Anti-Cancer Action

One of the mechanisms to explain the anticancer properties of LCn-3 PUFA involves the ability of these fatty acids to modulate the phosphorylation/activation of ERK1/2 and/or Akt and, in turn, the phosphorylation of multiple substrates involved in a variety of cellular responses and functions implicated in the development and progression of cancer. The modulatory activity of LCn-3 PUFA appears remarkable, since, as previously observed, in recent years the activation of both ERK1/2 and Akt has been intensively investigated in the oncologic preclinical and clinical setting, as they represent the ultimate targets of innovative therapeutic agents. In the case of the RAS/ERK pathway, these therapeutic agents mainly consist of inhibitors of the upstream GR Receptors (GFR, such as EGFR or HER/neu), RAF or MEK1/2 [44], and in the case of the phosphoinositide cascade, principally consist of inhibitors of GFR, as well as of PI3K. Recently, for their known antineoplastic properties and their ability to powerfully modulate both these signaling pathways in breast cancer, the potential of LCn-3 PUFA to function as adjuvants in combination with Trastuzumab has been investigated in in vitro and in preclinical models [26,45,46]. Trastuzumab is a specific inhibitor of the overexpressed receptor tyrosine kinase HER2/neu placed upstream of the activation of the Ras/ERK pathway [47]. Despite the ability of these fatty acids to modulate in an antineoplastic sense cancer apoptosis, cell differentiation, proliferation, migration, neo-angiogenesis, invasion, and metastasis in a variety of cancer cells being related in most cases to ERK1/2 or/and Akt inactivation, some results have demonstrated that LCn-3 PUFA may, on the other hand, drive their activation, thus suggesting that the mechanisms of their action may be tissue- or cancer-specific [48].

This review critically analyzes the results obtained in several experimental studies conducted in both in vitro and in vivo models of breast cancer, investigating the ability of these fatty acids in modulating ERK1/2 and/or Akt phosphorylation/activation. On the basis of these results, we will also discuss the potential complementary role of an LCn-3 PUFA treatment in combination with targeted therapies against breast cancer.

## 6. Evidence of an Inhibitory Effect of LCn-3 PUFA on ERK1/2 and/or Akt Phosphorylation/Activation

Hyperactivation of the Ras/ERK signaling was observed in half of breast tumors [49,50] and has been associated with malignancy and poor prognosis in breast cancer patients [51,52,53]. Most of the components in the RAS/ERK signaling pathway are overexpressed in the breast [54], but, in most cases their overexpression has not been related to activating mutation of either RAS or BRAF, as happens in other types of cancers, such as melanoma [55]. On the other hand, in order to explain the aberrant activation of this pathway, indirect mechanisms have been hypothesized, such as the involvement of other oncogenes or abnormal epigenetic regulation [49,51,56]. These results have led to the hypothesis that, regardless of the mechanism involved in the abnormal activation of the RAS/ERK signaling in breast cancer, the direct inhibition of ERK1/2, the ultimate target of this pathway, could represent the best option.

Several reports have shown that one mechanism through which LCn-3 PUFA inhibit breast cancer cell growth in vitro and in vivo involves the altered phosphorylation/activation of ERK1/2. Similarly, modifications in the expression/activation of Akt in breast cancer cells have been implicated in the powerful antineoplastic activities exerted by LCn-3 PUFA fatty acids. Akt represents the ultimate target of the IP3K/Akt signaling, which has been demonstrated to be the most frequently activated pathway in breast cancers [57], particularly in those characterized by aggressiveness and a poor clinical outcome. The PIK3CA gene mutation is the most frequently observed in this pathway, in particular in luminal breast cancers [36,57].

### 6.1. In Vitro Research and Preclinical Animal Models

#### 6.1.1. In Vitro Models

A number of experimental papers that we will critically analyze hereinafter have focused on the antineoplastic effects of LCn-3 PUFA in breast cancer cell lines or in animals, and associated them with their ability to alter the expression/activity of ERK1/2 and/or Akt. Most of them are in vitro studies performed on the scarcely differentiated MDA-MB-231 and MDA-MB-453 cell lines, representing the most used models of triple negative breast cancer (Figure 2), since they lack estrogen and progesterone receptors (ER and PR) and do not overexpress Her2 receptor. The results of these studies are valuable since, even though only 15% of all breast cancers are triple negative, the available and most widely used targeted therapeutic strategies, such as the anti-hormone therapies or Her2 receptor inhibitors, are not applicable in patients carrying this kind of breast cancer.

Other papers investigated BT-474 or SK-BR-3 breast cancer cell lines, which have features of higher differentiation as compared to the triple negative cell lines, since they express the HER2/neu receptor at aberrantly high levels. These cell lines are models for the HER2/neu positive class of breast cancer (Figure 2), and the patients carrying these kinds of breast tumors (about 15% of all breast cancers) can be therapeutically treated with inhibitors of the HER2/neu receptor. Lastly, results have also been obtained by studying the well differentiated MCF-7 cells, which, similarly to normal mammary cells, express both ER and PR and do not overexpress HER2/neu. This cell line is a widely used model for the most frequent class of breast cancer (about 65%–70% of all breast cancers) (Figure 2) because it is hormone-positive and, therefore, treatable with anti-hormone therapies.

#### 6.1.2. Triple Negative MDA-MB-231 and MDA-MB-453 Breast Cancer Cell Lines

The analyzed papers demonstrated that the treatments of the triple negative MDA-MB-231 or MDA-MB-453 breast cancer cells with EPA or/and DHA, at concentrations ranging from 30 µM to 100 µM, are able to inhibit cell growth and proliferation, as well as to enhance apoptosis or sensitivity to the toxic action of the chemotherapy treatment with docetaxel [58,59,60,61,62].

In these studies, triple negative MDA-MB-453 cells were incubated for one to five days to observe these antineoplastic effects. The results were related to a significantly decreased expression of phosphorylated Akt (p-Akt) [58,62], p-ERK1/2 [59], or of both these biomarkers often used to evaluate the level of activity of the TKR signaling activity [60]. In one case [62] the apoptosis induction was related to the DHA-induced lipid rafts internalization, and Akt displacement from these membrane microdomains through proteasomal or lysosomal pathways. This result is relevant, as intact rafts are necessary for Akt activation [63], and raft disruption may lead to Akt inactivation. Remarkably, the level of EGFR, one of the receptors driving the signaling ultimately leading to Akt activation, was also reduced in the lipid rafts of MDA-MB-231 treated with DHA. Consistently, the results recently obtained by Hopkins et al. [64] indirectly suggested that the observed EPA ability to inhibit LPA- and EGF-induced proliferation of both MDA-MB-231 cells and MCF-7 cells could be ascribed to the inhibitory action of EPA on the phosphorylation/activation of ERK1/2 and Akt, which, on the contrary, was markedly stimulated by LPA and EGF in these cancer cells. In line with all these results, Sato et al. [61] associated the DHA-induced inhibition of MDA-MB-453 cell growth with the decreased expression of p-Akt induced by this fatty acid. However, unlike all the other results analyzed so far, the authors [61] also found that DHA induced p-ERK expression in these cells, although they did not discuss the possible reasons for this quite surprising and conflicting result. It is worth pointing out that they [61] treated the cells with DHA in its non-esterified form, whereas the other authors [59,60]—who, on the contrary, observed that DHA decreased the expression of p-ERK in MDA-MB-231 cells—treated the cells with DHA-methyl esters. It is possible that the esterification of DHA could influence the level of absorption and incorporation of this fatty acid in cell membranes, and its capacity to negatively affect ERK in these extremely undifferentiated cells. Strikingly, however, the EPA- or DHA-induced decrease in p-Akt was observed in all the works analyzed, regardless of the form of fatty acids used (free fatty acids, FFA, in [58,61,62]; methyl ester in [60]).

#### 6.1.3. BT-474 and SK-BR-3 Breast Cancer Cells Lines Overexpressing HER2/neu

In other studies, (Figure 4) [26,65,66,67,68] the anti-neoplastic effect of LCn-3 PUFA was investigated on the BT-474 and SK-BR-3 breast cancer cell lines, which are models of HER2/neu positive breast cancer (Figure 2).

Interestingly, all the works demonstrated that DHA was able to induce a decrease in the expression of p-Akt and/or p-ERK1/2 or both p-ERK and p-Akt in these HER2/neu overexpressing breast cancer cell lines. This information is striking, since p-ERK and p-Akt are the biomarkers that are generally used in this kind of cancer to verify if a drug, targeting the aberrantly activated signaling starting from HER2, is inhibiting it or not. Therefore, these results strongly suggest that the dietary supplementation with DHA, which is able to decrease the level of the activated form of ERK1/2 and Akt, may have the potential to act as a complementary treatment in combination with drugs targeted against the signaling starting from the HER2 receptor. It is worth underlining the agreement among the results, as these studies were performed by using highly variable experimental conditions. The length of the DHA treatment ranged from 12 h to 4 d, and different concentrations and chemical forms of DHA were administered to the cultures. Multiple antineoplastic effects induced by DHA (cell death and viability, apoptosis, cell migration and invasion) were investigated, and always strictly related to the inhibiting activity exerted by the fatty acid on the expression p-ERK and/or p-Akt. For example, Mason et al. [26] found that BT-474 cell exposition to 50 and 100 µM DHA for four days markedly inhibited cell viability (by 30% and 70%, respectively). However, they did not investigate whether the effect of DHA was due to the inhibition of cell proliferation or induction of apoptosis. However, by using the XTT assay, Mouradian et al. [65] showed that, in the same breast cancer cells, 100 µM DHA reduced cell proliferation by about 50% at 48 h. On the other hand, Rescigno et al. [67] observed that DHA did not induce any relevant change in the distribution of SK-BR-3 cells in the cell cycle phases, but induced cell death, as demonstrated by an increase in the cell population in the sub G0/G1 cycle phase, which, however, may reveal DNA fragmentation associated with either necrosis or apoptosis. It is worth noticing that, in this case, the effect was observed at concentrations of DHA equal or higher than those used in the BT-474 cells, and with a comparable length of incubation (100 µM for 72 h, and 300 µM for 48 h). Also in these conditions, substantial reductions of SK-BR-3 cell viability were observed (by 30% and 50%, respectively). It is worth mentioning that the authors [67] used DHA complexed to bovine serum albumin (DHA–BSA), which has been reported to foster fatty acid incorporation into the cells [69]. This suggests that the use of lower concentrations of DHA in this form could also have allowed them to detect known and specific antineoplastic effects of DHA, such as the induction of apoptosis. As a matter of fact, a specific induction of apoptosis was observed by Sun et al. [68], who treated SK-BR-3 cells with 10 times lower concentrations of DHA–BSA (30 µM, 48 h treatment). Moreover, Li et al. [66] observed that 100 µM DHA–BSA complex administered to these cells, for just a short time (12 h), inhibited their ability to migrate and invade in vitro. In this case, the DHA–BSA complex was added to the culture medium together with 20 µM α-tocopheryl-succinate and 0.1% butylated hydroxytoluene in order to prevent cytotoxicity due to lipid peroxidation. Zou et al. [70] also used DHA (80 µM) complexed to BSA. They observed that the 72-h exposure of SK-BR-3 and BT-474 cells to DHA–BSA was able to downregulate p-HER2 and p-Akt, but dramatically decreased cell viability by 50% and 90%, respectively. Since, as previously described, apoptosis induction and downregulation of some phosphorylated components of the HER2 pathway (p-ERK and p-MEK) was obtained by Sun et al. [68] by exposing SK-BR-3 cells to a much lower concentration of DHA–BSA (30 µM) for a shorter incubation time (48 h), without any change in cell viability, it is possible to hypothesize that DHA, added as a BSA complex, may be highly effective, and that it would be advisable to decrease the DHA–BSA concentrations administered to the cells to levels which do not excessively reduce cell viability.

Recently, Mason et al. [26] have directly investigated the possibility that LCn-3 PUFA could increase the antineoplastic action of a specific targeted therapy in BT-474 breast cells. They treated the cells with 50 µM DHA combined with 10 µg/mL Trastuzumab, a specific inhibitor of the HER2 receptor, and found that the combination inhibited cell growth more efficiently than each single compound administered alone. Moreover, the DHA/Trastuzumab combination inhibited more than Trastuzumab alone (by 27% and 56%, respectively) both p-ERK1/2 and p-Akt expression (Figure 4). Interestingly, the administration of α-linolenic acid (ALA, 18:3n-3)—the metabolic precursor of DHA found at high levels in vegetable oil and nuts—inhibited cell growth, but was not able to induce apoptosis or increase the phosphorylation of ERK1/2 and Akt.

#### 6.1.4. Well-Differentiated MCF-7 Breast Cancer Cell Line

Significant comparisons have been recently made between the effect exerted by DHA on SK-BR-3 breast cancer cell line (overexpressing HER/neu receptor) and the more differentiated MCF-7 breast cancer cell line [67] (ER/PR positive and not overexpressing HER2/neu) (Figure 4 and Figure 5). It was found [67] that the DHA–BSA complex, at relatively high concentrations (100–300 µM), reduced cell viability and proliferation in MCF-7 cells (Figure 5) with a much lower efficiency than that observed in scarcely differentiated SK-BR-3 cells, which overexpress HER2/neu receptor (Figure 4). Moreover, the study demonstrated that the DHA–BSA complex was not able to increase the fraction of MCF-7 cell in the sub G0/G1 cycle phase (indicating an induced DNA fragmentation, i.e., cell death) with the same efficiency shown in the SK-BR-3 cell line (Figure 4). The observation that the DHA–BSA complex inhibited p-ERK in MCF-7 cells at a much lower extent than in SK-BR-3 suggests that the dimension of the inhibitory effect on p-ERK is strictly related to the overexpression of HER2/neu, present only in SK-BR-3 cells. These results suggest that a complementary treatment with DHA combined with drugs targeting Ras/ERK and/or phosphoinositide signaling pathways could be particularly promising in less differentiated breast cancers overexpressing the HER2/neu receptors. However, Sun et al. [71] had previously demonstrated that, even at a much lower concentration, DHA–BSA (30 µM) was able to efficiently induce apoptosis in the same MCF-7 cell line through the inhibition of the ERK1/2 signaling pathway (Figure 5). Further support to the hypothesis that the pro-apoptotic effect of LCn-3 PUFA in MCF-7 cells could involve the modulation of the ERK and Akt signaling was provided by Cao et al. [72], who, however, used quite a high concentration of both EPA and DHA (90 µM) as FFA acids. Interestingly, they also found that both EPA and DHA shifted the pro-survival effect exerted by 17 β-estradiol (E2) on MCF-7 cells to a pro-apoptotic effect, and that the pro-apoptotic effect of E2 did not occur through the typical nuclear estrogen receptors, but through the activation of the G protein coupled estrogen receptor 1 (GPER1). They demonstrated that the induction of this E2-driven response by LCn-3 PUFA was related to the suppression of both ERK and Akt phosphorylation. More recently, Chen et al. [73], who also investigated the effect of DHA on MCF-7 breast cancer cells, used quite high concentrations of DHA (100–200 µM, given as FFA for 20 h), followed by an additional incubation with 12-*O*-tetradecanoylphorbol-13-acetate (TPA) that lasted an additional 24 h. They described the ability of this fatty acid to inhibit TPA-induced MCF-7 cell migration and invasiveness in vitro. However, unlike Rescigno et al. [67], who found that these high concentrations of DHA–BSA exerted a marked reducing effect on cell viability, Chen et al. [73] observed no changes in cell viability in all the experimental conditions employed. This may be due to slightly different experimental conditions, such as the form of DHA used; in the study by Chen et al. [73] it was added to the cells as FFA, and probably not so efficiently transported into the cells as DHA complexed with BSA. The inhibitory effect exerted by DHA on the TPA-induced cell migration and invasiveness [73] was associated by these authors with the ability of this fatty acid to suppress MMP-9 expression and activity. In agreement with the previously reported involvement of MAPK and phosphoinositide signaling pathways in TPA-mediated MMP-9 expression [74,75], the authors observed that DHA also reduced ERK1/2 and Akt phosphorylation. The first among all the studies identified and analyzed in this review [76] had also been conducted on MCF-7 cells, which, however, were transfected to overexpress constitutively active Akt. The treatment for 24 h with EPA- or DHA-FFA inhibited the insulin-induced increase in activated p-Akt, but EPA was particularly efficient, with its effects already visible at 20 µM and reaching the maximum at 200 µM. The authors also demonstrated that the addition of 40 µM EPA made MCF-7 cells overexpressing active Akt more responsive to the growth-inhibiting action of the antiestrogen Tamoxifen. These early findings are still very promising, since, as reported above, the activation of Akt is one of the mechanisms involved in breast cancer resistance to Tamoxifen. Moreover, they demonstrate that, using non-cytotoxic concentrations of LCn-3 PUFA, which are not able to produce a maximal growth inhibiting effect (40 µM in this case), may represent a better option when studying the molecular effects or combinations of these compounds with other antineoplastic agents.

#### 6.1.5. Considerations on the Highly Variable Experimental Conditions Used in the In Vitro Studies

It is evident that the extremely different experimental conditions used in the in vitro studies may influence the antineoplastic efficiency of LCn-3 PUFA, and make it difficult to draw general conclusions from the results obtained. There is one aspect concerning the experimental conditions used in the in vitro studies with these fatty acids that is often overlooked, but that deserves more attention. Most of the authors have used DHA in their experiments. Although, to obtain the desired effects, the different authors have used highly variable concentrations of this fatty acid (ranging from 30 to 300 µM), they often claim to have used concentrations of DHA that are similar to those physiologically found in human serum [67,68]. Actually, a variable value of 150–300 µM (and even more) has been often reported as the normal concentration of DHA present in the serum of different populations around the world, with different nutritional intakes of LCn-3 PUFA [77,78,79,80]. These values correspond approximately to the level of the fatty acid esterified to serum phospholipids (PL) (DHA-PL), which is considered a useful biochemical index for DHA status and a marker for fish intake of EPA/DHA [81,82,83]. However, it is also possible to refer to the serum level of non-esterified fatty acids (DHA-NEFA), deriving mostly from dietary triglycerides stored in adipose tissue and then released, and whose value is basally about 10 times lower than that of DHA-PL. In a human study, Conquer and Holub [84] reported that, starting from a basal level of DHA-NEFA and DHA-phospholipids (DHA-PL) of about 1 µM and 150 µM, respectively, the levels of serum DHA-NEFA and DHA-PL increased to about 12 µM and 400 µM, respectively, following a six-week supplementation with 1.5 g/day DHA given as the algal-derived triglyceride DHASCO [84]. It is worth underlining that the most widely accepted beneficial health effects of LCn-3 PUFA, i.e., those reported against the development of some cardiovascular pathologies [84], has been generally associated with the LCn-3 PUFA-NEFA serum level. Unlike FA esterified to PL, which travel throughout the circulation included in lipoproteins, NEFA travel bound to albumin, which has been reported to make the uptake of NEFA by cells and tissues easier [69]. Thus, it is difficult to establish the physiologic concentration that would be more advisable to use in the in vitro studies. However, in our opinion, and in addition to these considerations, highly cytotoxic concentrations of DHA able to reduce cell viability by more than 90% should be avoided when cellular processes (e.g., apoptosis or cell cycle progression), as well as signaling pathways, are under study. Moreover, in the search for an optimal and physiological concentration of LCn-3 PUFA for the in vitro studies, it is worth bearing in mind that, as previously highlighted, cell viability may also be affected in highly variable ways by the chemical forms of PUFA (i.e., unesterified fatty acid or the DHA–BSA complex), even if identical concentrations are used. That may not only be related to the easier cellular uptake of FA bound to albumin [69], but also to the well-known antioxidant effect of albumin, which may protect against LCn-3 PUFA-induced cytotoxicity [85,86].

In conclusion, if the researchers’ aim is to investigate the specific effect of LCn-3 PUFA on cellular processes and/or signaling pathways/molecules, and not to induce high levels of cytotoxicity that could compromise and alter the cellular responses, our recommendations are:

(1) A range of LCn-3 PUFA concentrations should be tested for each single cell line (for most cells usually not higher than 50 µM), as well as variable lengths of incubation, thus trying to avoid those conditions that induce highly cytotoxic effects (i.e., a reduction of viability higher than 10%) for further experiments;
(2) It should not be considered appropriate to justify the use of relatively high concentrations of LCn-3 PUFA only on the basis of their already known physiological serum concentrations in humans in vivo, if these concentrations, when administered in vitro, cause a dramatic reduction of cell viability;
(3) In vitro administration of LCn-3 PUFA to cells, either in the form of FFA or the FA–BSA complex, should be considered equivalent, provided that the doses used are not highly cytotoxic during incubation. However, it is expected that FA–BSA complexes may affect cell processes and/or signaling pathways/molecules more efficiently than comparable concentrations of LCn-3 PUFA in the form FFA.

#### 6.1.6. Animal Models

Recent studies performed in vivo by Mason et al. have yielded interesting suggestions regarding the potential adjuvant role of LCn-3 PUFA in HER2/neu positive breast cancer [45,46]. The authors injected BT-474 cells into nude mice fed with a diet enriched with 4% Flaxseed oil (FSO) for four weeks, and combined this diet with Trastuzumab, an antibody specifically directed against the overexpressed HER2/neu receptor, and currently used in the therapy of human breast cancer positive for this receptor. The design of the work was related to previous preclinical works published by the same group [87,88], demonstrating the inhibitory effect of an FSO diet on the growth and survival of MCF-7 cells transplanted in nude mice. They had also shown that FSO effects were related to the reduction of ERK- and GF-mediated signaling pathways [88]. The FSO used by these authors contained 57% of α-linolenic acid (ALA, 18:3n-3), which is the metabolic precursor of DHA, but is not efficiently converted into DHA in mammalian cells. Indeed, Mason et al. [46] found that the FSO diet increased ALA levels in BT-474 tumors by about eight times, but the levels of DHA were less than doubled [46]. In this case, dietary FSO increased the suppressing effect of Trastuzumab on tumor growth by interacting with Trastuzumab to suppress p-HER2, p-ERK-2, and p-AKT1 expression [46]. However, as reported above, in their most recent in vitro work, Mason et al. [26] found that, whereas DHA was able to increase the suppressive action of Trastuzumab on p-ERK and p-Akt expression, such a potentiating effect was not found when they tested ALA. Therefore, in our opinion, it would be useful to perform further in vivo experiments by directly supplementing mice with DHA combined with Trastuzumab.

The findings obtained by Chen et al. [89] were also of great interest. They used the mouse mammary tumor virus (MMTV)-neu(ndl)-YD5 model overexpressing HER2 and bred these transgenic mice with the fat-1 mice, which are capable of endogenous n-3 PUFA synthesis. In the offspring they found a reduced growth and multiplicity of developed tumors. This finding strongly suggested the involvement of n-3 PUFA in the regulation of the HER-2 pathway. In light of this, Zou et al. [70] injected HER-2 positive E0771 mouse breast cancer cells in fat-1 mice, and investigated how the tumor HER-2 signaling pathways could be influenced by the endogenous high level of LCn-3 PUFA of fat-1 mice. In line with the results obtained by Chen et al. [89], these authors found reduced in vivo growth of the breast cancer cells injected in fat-1 mice, and an inhibition of HER-2 pathway, demonstrated by the marked decrease of HER2 expression in the tumor tissues growing in fat-1 mice.

In agreement with the above analyzed studies, Sun et al. [68] also showed that the phosphorylation of MEK and ERK was lower in the fat-1 transgenic mouse mammary tissue than in wild-type tissue. They also found that BAD was less phosphorylated, and able to positively regulate apoptosis [68]. These results suggest that, in normal mammary tissue, the LCn-3 PUFA, through their ability to maintain the ERK1/2 and PI3K/Akt/mTOR at low level of activities, could have the potential to exert a preventive action against the risk of breast cancer, comparable to that of selective estrogen receptor modulators, now approved for breast cancer prevention (Tamoxifen and Raloxifene). Moreover, by using a rat model of *N*-Methyl-*N*-nitrosourea (MNU)-induced mammary tumorigenesis, also Jiang et al. [90] confirmed the results of the in vitro studies analyzed in the previous paragraph. They found that feeding the rats subject to mammary carcinogenesis a diet at high n-3/n-6 ratio, apoptosis was induced and the phosphorylated forms of IP3K, Akt, and mTOR were downregulated in the developed tumors. Interestingly, they also observed that the diet at a high n-3/n-6 ratio induced in the tumors the expression of both AMP Activated Protein Kinase (AMPK) and phospho-Raptor, which are in the arm of this pathway associated with the suppression of mTOR signaling. Confirming and extending these results, Chen et al. [89] found that a diet with high levels of LCn-3 PUFA induced apoptosis in the tumors developed in MNU-treated rats by downregulating both p-ERK1/2 and p-Akt. The LCn-3 PUFA-induced inhibition of Akt phosphorylation was convincingly demonstrated by the same authors [89] also by using additional animal models of breast cancer: (a) nude mice implanted with fat-1 expressing MDA-MB-231 breast cancer cell lines; (b) fat-1 transgenic severe combined immune deficiency (SCID) mice implanted with breast tumor cells; and (c) fat-1 transgenic mouse mammary tumor viruspolyoma virus middle T oncogene double-hybrid mice.

#### 6.1.7. Contrasting Results

Despite all the above reported in vitro and in vivo results, which together demonstrate the inhibitory effect of LCn-3 PUFA on ERK and Akt activation, Wu et al. [91] have recently reported that the treatment of MCF-7 breast cancer cells in vitro with high doses of EPA or DHA (100 and 200 µM) attenuated apoptosis induced by the anti-estrogenic drug Tamoxifen, and induced p-ERK and p-Akt expression. These findings are of great practical importance and deserve great attention, since Manni et al. [92,93] had previously observed that an FO diet rich in LCn-3 PUFA enhanced the chemopreventive effects of Tamoxifen on MNU-induced rat mammary carcinogenesis by inhibiting the development of preneoplastic lesions, as well as the regression of those already established. On the other hand, the results obtained by Wu et al. [91] on MCF-7 cells in vitro suggest that LCn-3 PUFA may interfere with the antineoplastic effect of Tamoxifen in breast cancer cells. Indeed, increased ERK1/2 and Akt activation were previously reported to induce resistance to Tamoxifen in breast cancer [94,95]. However, in our opinion, there are some critical points regarding the experimental conditions used in vitro by these authors that should be critically analyzed. In the work by Wu et al. [91], the high LCn-3 PUFA concentrations (100 and 200 µM) able to suppress MCF-7 cell apoptosis [91], also caused a dramatic decrease in cell viability (for instance, treatment with 100 and 200 µM DHA for 24 h caused a decrease of viability by about 50% and 90%, respectively). In our opinion, and as previously underlined, it would be advisable to avoid these extremely cytotoxic concentrations to evaluate the effects of these fatty acids on complex cellular processes, such as apoptosis, or on the molecular pathways regulating these processes, including those ultimately leading to ERK and Akt activation. Interestingly, however, when these authors [91] administered Tamoxifen in combination with lower LCn-3 PUFA concentrations (5 and 50 µM), that reduced MCF-7 cell viability to a much lower extent (treatments with 5 or 50 µM DHA for 24 h: about 25% viability decrease in both cases); apoptosis was increased and not suppressed. Moreover, 50 µM DHA induced a significant increase of p-ERK1/2 and p-Akt expression, but only after very short incubation times and transiently (between 30 min and 12 h for p-ERK1/2, and at 12 h for p-Akt). These effects were lost when the treatments with DHA were prolonged to 24 h. These findings strongly suggest that longer periods of incubation with LCn-3 PUFA (24 h–5 days), comparable to those used in all the other in vitro studies herein analyzed, would be needed to obtain the suppression of cell growth and proliferation or induction of apoptosis, as well as a stable and long-lasting inhibition of p-ERK and p-Akt expression. Moreover, these stable effects obtained with long in vitro incubation times were also obtained in the animal mammary tissues examined in the in vivo studies analyzed in the present work [45,68,70,89,90]. Similarly, in our opinion, longer periods of incubation would be also appropriate to evaluate the effects of LCn-3 PUFA/Tamoxifen combination on the expression of p-ERK1/2 and p-Akt. On the contrary, Wu et al. [91] limited their observations to a very short period (6 h), and used a concentration of DHA (100 µM) that, under the experimental conditions used, yielded a strong cytotoxic effect [91]. In these conditions, the combination DHA/Tamoxifen induced a significant increase of p-ERK1/2 and p-Akt expression compared to the control condition without either DHA or Tamoxifen. In our opinion, however, these results still need to be confirmed through combination experiments by longer incubation times, lower DHA concentrations, and also comparing the effect of the DHA/Tamoxifen combination to the effect of each of the two antineoplastic agents administered alone.

### 6.2. Human Interventional Studies

Recently Fabian et al. [96,97] demonstrated that LCn-3 PUFA produced a preventive action against the development of breast cancer in women through the regulation of HER2, Akt and mTOR. Their two phase II single-arm pilot studies were conducted in pre-menopausal [96] and post-menopausal women [97] showing cytological evidence of hyperplasia ± atypia in the biopsies obtained at baseline by fine needle aspiration in the periareolar area. The patients received a daily dose of 3.4 g EPA + DHA ethyl esters for 6 months, and, in both studies, the LCn-3 PUFA treatment induced a clear antiproliferative effect (indicated by the decreased level of the marker Ki-67 in the biopsies). Moreover, the treatment also significantly decreased the cytomorphology index score in the postmenopausal women. The protein array analysis performed on serum and mammary tissue samples revealed that these antineoplastic effects were associated in post-menopausal women with favorable modulation of several proteins involved in mitogen signaling and cell-cycle arrest, including HER2. However, no obvious effects on proteins downstream of mTOR, included p-Akt, were found in this case. In the pre-menopausal women, the proteomic analysis of the specimens revealed mixed effects on protein involved in the AKT/mTOR pathways, with increases in PDK1 (the protein kinase that specifically phosphorylates Akt on Thr308 downstream PI3K) and AktpS473. However, there was a decrease in other proteins necessary for the mTOR proto-oncogenic signaling (i.e., Raptor, cyclin D1, PRAS40, and IF4E), thus suggesting that LCn-3 PUFA were also able to induce the suppression of the mTOR-associated cell growth and proliferation. In order to explain their varying results, the authors suggested that, since the biopsies were performed after a protein-rich meal, the mixed changes in proteins associated with the AKT/mTOR pathway were possibly related to the fact that the amino acid load could have caused an increase in the mTOR pathway related to insulin signaling/sensitivity. These single-arm studies are of great interest, but further placebo-controlled trials designed on the basis of the results previously obtained could provide additional information on the role that LCn-3 PUFA may exert in the primary prevention of breast cancer. Moreover, the tissue sampling should be performed in controlled nutrient intake conditions, to better study the changes in the expression of proteins associated with the Akt/mTOR pathway branch implicated in the regulation of cell growth and proliferation, and not with the branch related to the insulin signaling and activated after a protein-rich meal. Ultimately, lower and safer doses (of about 2–3 g/day) of LCn-3 PUFA could be tested in the patients, since it was observed that such doses are already able to lead to the maximal increase (about 2–3-fold average increase) in LCn-3 PUFA incorporation in serum phospholipids and in tissues [98,99,100].

## 7. Conclusions

During the last few years numerous in vitro and in vivo preclinical studies have demonstrated that dietary LCn-3 PUFA have the potential to exert a preventive and therapeutic role against breast cancer through the negative regulation of the ERK and/or phosphoinositide survival pathways. These findings strongly suggest the potential use of LCn-3 PUFA as preventive dietary compounds, or adjuvants in combination with therapies directed to single targets of these pro-survival pathways, which have been proven to induce escape mechanisms and resistance in cancer cells [16]. In particular, our analysis of the animal studies has shown that there is a general agreement on the powerful role of LCn-3 PUFA as negative modulators of the ERK and phosphoinositide survival pathways, thus making these results an important basis for future human studies.

Moreover, we have shown that almost all the in vitro studies analyzed corroborate the same hypothesis, although they were performed on different breast cancer cell lines with a variable degree of differentiation, and exposed to highly variable doses of LCn-3 PUFA in different chemical forms (as FFA or bound to albumin). However, some interesting suggestions stemming from these in vitro studies require further examination, such as the hypothesis put forward by Rescigno et al. [67]. This hypothesis indicates that the scarcely differentiated breast cancer cells overexpressing HER2/neu receptor could be the most sensitive to the negative modulation exerted by LCn-3 PUFA on these survival pathways [67]. The hypothesis, very important for its potential and practical consequences, in our opinion, should be further investigated by exposing breast cancer cells to concentrations of PUFA lower than those used in this study (100 and 200 µM), which, in the overexpressing HER2 SK-BR-3 cells, dramatically affected cell viability (by 20%–30% and 40%–70%, respectively, after 24–72 h) [67]. Lower concentrations of LCn-3 PUFA would also be required to confirm other studies performed with highly cytotoxic concentrations. Some of these experiments are particularly interesting since they are performed with combinations of LCn-3 PUFA and estrogens or anti-estrogens, such as the one by Cao et al. [72]. This work suggests the possibility that LCn-3 PUFA may exert a preventive action against breast cancer by converting the pro-survival activity of estrogens into a pro-apoptotic effect by blunting the ERK and Akt signaling [72]. A second study [91] also deserves further attention since, differently from all the other reports analyzed, it showed that very high and cytotoxic doses of LCn-3 PUFA were able to attenuate apoptosis induced by the anti-estrogenic drug Tamoxifen in breast cancer cells, and that the effect was related to the very early and transient increase of p-ERK and p-Akt expression observed.

Lastly, from our review of the literature, we have found that only two preliminary human single-arm studies [96,97] have so far investigated the hypothesis that LCn-3 PUFA may play a preventive and therapeutic role against breast cancer through the modulation of the pro-survival signaling pathways under study. These studies were performed by analyzing tissues from women at high risk of breast cancer who received a supplement consisting of quite a high dose of LCn-3 PUFA. However, although on the whole these studies have provided promising outcomes, quite varied results were obtained relating to the ability of these fatty acids to modulate the mTOR proto-oncogenic signaling downstream the activation of Akt. As the authors suggested in their papers, this was probably correlated to the confounding nutritional patient conditions when the mammary biopsies were performed (after the intake of a high protein meal). Therefore, these studies also need further confirmation by placebo-controlled trials performed by using well-controlled nutritional conditions.
